# Before and after case reporting: A comparison of the knowledge, attitude and practices of the Jordanian population towards COVID-19

**DOI:** 10.1371/journal.pone.0240780

**Published:** 2020-10-15

**Authors:** Eman Elayeh, Shereen M. Aleidi, Rawan Ya’acoub, Randa N. Haddadin

**Affiliations:** 1 Department of Biopharmaceutics and Clinical Pharmacy, School of Pharmacy, The University of Jordan, Amman, Jordan; 2 Department of Pharmaceutics and Pharmaceutical Technology, School of Pharmacy, The University of Jordan, Amman, Jordan; Jouf University, Kingdom of Saudi Arabia, SAUDI ARABIA

## Abstract

Coronavirus disease- 2019 (COVID-19) is an emerging contagious infectious disease. It is pandemic and has affected more than 21 million people and resulted in more than 750,000 deaths worldwide (https://www.worldometers.info/coronavirus/#countries; 14/08/20). Our research group initiated a study to ascertain the knowledge, attitude and practices (KAP) of Jordanians toward COVID-19 prior to any initial case report in Jordan. This project was underway when the first Jordanian case was reported. We extended our study to identify how case reporting would alter public KAP towards COVID-19. This cross-sectional study randomly selected and recruited 2104 Jordanian adults. A four-section questionnaire was devised to address the sociodemographic characteristics of the subjects and their KAP toward COVID-19. The mean knowledge score for the study population was 15.9 ± 2.2 (out of the 20 knowledge questions), with 60.9% of the participants having good knowledge about COVID-19. Participants’ practices to prevent transmission of COVID-19 were adequate in more than 60% of participants. Most participants had positive attitudes regarding their role in preventing COVID-19 and many of the participants’ attitudes and practices changed to more appropriate ones after reporting the first case of COVID-19 in Jordan. The percentage of participants who trust the government in confronting COVID-19 increased significantly (p value < 0.001). However, one alarming and unexpected finding was that the prevention practice score of participants working in the medical field was similar to those from the general population. This may necessitate stricter training and guidelines for this group who will be in the frontline in combating the disease. Impact of this study: The data generated from this study shows that when cases of disease were reported, the public’s attitudes and practices improved in many aspects, and that confidence in the government to contain the disease was boosted. We believe that this study is important in allowing other, international governments to develop an understanding of public KAP during pandemic disease outbreaks.

## Introduction

Coronavirus disease (COVID-19) is a contagious infectious disease caused by a novel Coronavirus 2019-nCoV, also known as Severe Acute Respiratory Syndrome Coronavirus-2 (SARS-CoV-2) [1, 2]. This virus has not been previously identified in humans, and was first discovered in Wuhan, China, in December 2019. The SARS-CoV-2 primarily has been shown to attack the human respiratory system, causing symptoms ranging from mild flu-like symptoms to severe damage to the respiratory system (and sometimes other organs), and death [3]. Elderly people and those with chronic medical conditions are the most vulnerable to the complications of COVID-19 [2, 4]. COVID-19 has disseminated rapidly to all continents in a short time, with an exponential rate of incidence [5, 6]. Due to this high rate of transmission and disease incidence, the World Health Organization (WHO) declared COVID-19 a global pandemic that presents a major threat to the humanity on 11^th^ March 2020 [4]. To date (https://www.worldometers.info/coronavirus/#countries; 14/08/20), it has occurred in more than 213 countries with a total number of confirmed cases that surpassed 21,100,000, with more than 750,000 deaths. Currently, there is no specific effective treatment or vaccine available for COVID-19. Many ongoing clinical trials are investigating the effect of different suggested medications including anti-viral drugs [7] and vaccines. At the time of writing, several candidate vaccines are completing Phase 3 trials and it is hoped that a viable vaccine may be available before the end of 2020 [8]. Therefore, the only current way to avoid the disease is to prevent the virus transmission. The whole regarding virus transmission is so far not complete and the information about COVID-19 is constantly evolving. It has been indicated that COVID-19 is transmitted directly by close contact (within 1 meter) with an infected person through respiratory droplets (>5–10 μm) and also through touching the surfaces contaminated with infected droplets [9]. This is reflected in the WHO recommendations for viral transmission, which include self-hygiene, physical distancing of at least 1 meter from infected patients, avoiding crowded places, covering mouth and nose when coughing or sneezing, intensive and regular washing of hands with alcohol based hand rub or with soap and water for at least 20 seconds, maintaining a good ventilation for homes and indoor settings and the appropriate use of masks in public places and public transport [10].

Jordan is a developing country in the Middle East with a population of approximately 10 million. In the last few years the country has seen a massive influx of more than a million Syrian refugees, which comprises along with other refugees (eg: Palestinian, Iraqi, and others), around one third of the population (approximately 3,000,000 refugees) [11]. In a country with limited resources, this has placed large constraints on the national infrastructure and on the health sector in particular [12, 13]. Governmental understanding of these demands on resources, especially in the healthcare sector, led them to adopt strict control measures on the international occurrence of COVID-19 cases. These measures were escalated according to the degree of spread of the disease in neighboring countries and around the world. The measures were implemented even before reporting any cases in the country. The immediate measures were (i) closing schools and universities, (ii) compulsory quarantining of all incoming international passengers in hotels, (iii) general self-quarantine of the population, and (iv) the partial closure of land and sea borders and resultant closure of all the borders including airports [14, 15]. During this period, the Jordanian Ministry of Health (MOH) used the media to educate the public about the disease, its mode of transmission, the importance of social distancing, washing hands, using disinfectants, home hygiene and advising people to avoid any gatherings (including the closure of all mosques and churches).

After reporting 70 cases which were all isolated in dedicated hospitals, the Jordanian Government applied a nationwide lockdown and activated the Defense Law, which allows the army and police to ensure the adherence of public to the curfew [16].

Under these circumstances, where public health is threatened by the pandemic and since the people’s attitudes and practices play a crucial role in limiting or spreading the disease in their community, evaluating the knowledge, attitude, and practice (KAP) of the general public towards the COVID-19 is crucial. Therefore, the initial aim of this study was to evaluate the overall knowledge, attitude and practices (KAP) of Jordanian citizens to the ongoing international situation regarding the emergence and pandemic nature of COVID-19. However, we were lucky that our study was almost complete when the first case of the disease was reported in Jordan. Hence, we rapidly adapted our study in order to engage more participants and to evaluate how this reported case altered the KAP of Jordanian citizens. The aim of this report is to aid decision makers in their understanding of the public knowledge and awareness of the disease, the attitudes of the public and their practices to take necessary measures to prevent disease spread.

## Materials and methods

### Study design and data collection

This was a cross-sectional study conducted during the early days of COVID-19 pandemic of 2019 and 2020 and over the period from 12^th^ February to 19^th^ March 2020. When the study started there were no reported cases in Jordan. During the study, the first COVID-19 case was reported in Jordan, which encouraged the authors to extend the study and include more participants in order to identify how case reporting would alter the public KAP towards COVID-19. The study targeted adult resident Jordanian nationals. It was a questionnaire based and self-selection study. The Institutional Review Board (IRB) at the Deanship of Scientific Research, The University of Jordan, approved the study protocol and the questionnaire (IRB 28/2020).

The questionnaire was developed based on extensive literature review [3, 17–20]. It consisted of four main sections; (a) demographic and general characteristics of the participants, (b) participants knowledge regarding COVID-19, its mode of transmission, and its preventative measures (20 questions), (c) participants’ attitudes towards COVID-19 and its preventative measures (13 questions), and (d) participants’ practices towards prevention of COVID-19 transmission (6 questions). The questionnaire content was translated from English into Arabic and then critically revised and face-validated by several academic colleagues. Since the Jordanian general public knows COVID-19 as *“coronavirus disease”*, this term was used in the questionnaire. Amendments were made according to the notes and comments received after piloting to a sample of 50 adults chosen from the friends, neighbors and coworkers of the authors. The questionnaire was then developed as a Google form and disseminated to participants via mobile applications, eg: WhatsApp [21, 22]. Snow ball sampling technique was used to distribute the Google form and enroll participants [22, 23]. The link to the Google form was sent to the contact lists of the authors, in addition to neighbors, friends, relatives, coworkers of all levels and departments, friends of friends, different WhatsApp groups, who were also asked to complete the form and to disseminate it to all the people they know in Jordan. This method of disseminating the questionnaire was selected in view of the need to reduce direct contact with participants under the pandemic situation. Participants were asked not to identify themselves in any fashion. Also they were informed that completing the questionnaire and returning it were considered as a formal consent to participate in the study since the participant can, at any moment, stop answering questions or choose not to *“submit”* the final form.

### Study sample size

A minimum sample of 1050 adults aged 18 and older was estimated based on the following equation [24]: $\text{N}=\frac{PQ{\left(Z_{\alpha }+Z_{\beta }\right)}^{2}}{d^{2}}$, where N is the sample size, Zα: type one error = 1.96 when α = 5%; Zβ: type two error = 1.28 when β = 10%; Q = 1-P: expected non-prevalence; P = proportion in the population possessing the characteristic of interest (based on the estimate that 50% of the respondents knew general information about COVID-19, its routes of transmission and the main preventative measures), d = one-half of the desired interval of confidence, in this study d = 5%.

Accordingly, by filling in the equation, N = 0.5x0.5 (1.96+1.28)^2^/ 0.05^2^ = 1049.76.

### Statistical analysis

Statistical analysis was performed using SPSS version 20.0 (SPSS Inc., Chicago, IL). Descriptive statistics were used to describe demographic characteristics of participants. Categorical variables were presented as valid percentages to account for missing data with their frequencies, while continuous variables were presented as mean with standard deviation (SD).

Two scores were calculated for the participants: knowledge about COVID-19 and its preventative measures, and participants’ practices towards preventing transmission of COVID-19.

Participants' knowledge scores were evaluated using the number of correct questions they answered out of 20 COVID-19 knowledge questions. In order to calculate the knowledge score, correct answers were assigned a score of one, while wrong or *“I don’t know”* answers were assigned a score of zero. Participants were considered adequately knowledgeable if their knowledge score, for all knowledge questions, was higher than or equal to the sample median of the knowledge score.

Internal consistency of the knowledge questions was tested using Cronbach alpha coefficient as a reliability test. The results showed that the Cronbach alpha for knowledge (20 items) was 0.7. Cronbach alpha within the range 0.6 to 0.7 is considered adequate and reliable [25].

Similarly, participants practice towards preventing transmission of COVID-19 score was calculated by assigning a score of one for appropriate practices, while inappropriate practices were assigned a score of zero. The maximum value of practice score is six based on the total number of correct practice items. Participants’ knowledge, attitudes and practices were compared before and after the reporting of the first confirmed case of coronavirus in Jordan which was documented on 2^nd^ March 2020. By default, ‘Google forms’ identifies the date and time of each participant’s response which enabled us to sort the responses submitted into those made before and after case reporting. Chi-square test was used for these comparisons. Parametric tests including independent sample t-test and one way ANOVA test were used to test the differences among the variables that affect both knowledge and practice scores (bivariate analysis) as specified in the results section (data were first tested for normality using Kolmogorov-Smirnov (K-S) test and for homogeneity of variances using Levene’s test). In addition, independent sample t-test was used to test the differences in the scores between the two groups (before and after case reporting).

All hypothesis testing was two-sided. A *P*-value of < 0.05 was considered significant.

## Results

### Sociodemographic characteristics of participants

The total number of participants recruited in the study was 2104. 832 participants were recruited before case reporting and 1272 participants were recruited after case reporting. More than 50% of participants were in the age group of 18–35 (62.7%, n = 1321). Females accounted for 75.4% (n = 1586) of the study population. More than 75% of participants had a university or postgraduate degree (81.6%, n = 1717), and 43·6%, (n = 917) had education in the medical field. Since the cohort participating in the study after case reporting differed from those participating before case reporting, there were variations in the sociodemographic data of the two groups (p< 0.05, Chi square) except for gender and working in the medical field which were similar (p>0.05). Participants' sociodemographic characteristics are presented in Table 1.

**Table 1:** Sociodemographic characteristics of all participants (n = 2104) including those participated before case reporting and after case reporting.

Variables	All participants (n = 2104) % (*n*)	Before (n = 832) % (*n*)	After (n = 1272) % (*n*)
**Age group (years)**			
18–25	30.9 (651)	28.4 (236)	32.6 (415)
26–35	31.8 (670)	28.1 (234)	34.3 (436)
36–45	18.8 (395)	24.0 (200)	15.3 (195)
46–55	11.6 (244)	13.6 (113)	10.3 (131)
>55	6.8 (144)	5.9 (49)	7.5 (95)
**Gender**			
Male	24.6 (518)	24.2 (201)	24.9 (317)
Female	75.4 (1586)	75.8 (631)	75.1 (955)
**Marital status**			
Married	54.8 (1152)	58.3 (485)	52.4 (667)
Single	42.5 (894)	38.8 (323)	44.9 (571)
Others	2.8 (58)	2.9 (24)	2.7 (34)
**Educational level**			
Post graduate	16.4 (346)	20.4(170)	13.8 (176)
University level	65.2 (1371)	64.7 (538)	65.5 (833)
Diploma	8.7 (184)	8.8 (73)	8.7 (111)
12 years or less	9.6 (203)	6.1 (51)	11.9 (152)
**Employment status**			
Working	54.6 (1149)	57.0 (474)	53.1 (675)
Not working	25.8 (543)	19.7 (164)	29.8 (379)
Retired	6.1 (128)	5.9 (49)	6.2 (79)
Student	13.5 (284)	17.4 (145)	10.9 (139)
**Education in the health or medical field (yes)**	43.6 (917)	49.5 (412)	39.7 (505)
**Medical field specialty**			
Medicine or dentistry	5.4 (113)	14.8 (72)	6.2 (41)
Pharmacy	24.2 (510)	34.8 (170)	51.8 (340)
Nursing	5.0 (106)	15.4 (75)	4.7 (31)
Medical labs	2.4 (51)	5.1 (25)	4.0 (26)
Others	17.3 (364)	29.9 (146)	33.2 (218)
**Work in the medical field (yes)**	33.7 (710)	34.6 (288)	33.2 (422)

### Knowledge about COVID-19

The mean knowledge score for the whole study population was 15.9 ± 2.2 (out of the 20 knowledge questions), with 60.9% (*n* = 1281) of the participants having a score equal to or higher than the median (16.0) and consequently were considered to have adequate knowledge about COVID-19. Only 2.4% (n = 51) of the participants were able to correctly answer all of the 20 knowledge questions and 8% (n = 168) of participants were able to correctly answer 19 questions. In addition, 17% (n = 357) of participants knew the general information related to the COVID-19, its symptoms and the highest risk group. However, more than 50% of the study population incorrectly thought that death is a common complication of COVID-19 (44.2%, n = 929) (Table 2).

**Table 2:** Participants' Knowledge regarding COVID-19, its mode of transmission, and its preventative measures.

Question	Total number (n = 2104)	Before (n = 832)	After (n = 1272)	P value
	Correctly answered % (*n*)			
**Definition, signs and symptoms, risk groups and complications [mean score ± SD]**	**[5.3±1.2]**	**[5.1 ±1.2]**	**[5.5 ±1.2]**	**<0.005**
Coronavirus is a new virus that first appeared in Japan 2019. *	59.2 (1246)	55.2 (459)	61.9 (787)	0.009
Coronavirus mainly affects the respiratory system.	93.9 (1976)	93.4 (777)	94.3 (1199)	0.039
Coronaviruses are zoonotic, meaning they are transmitted between animals and people.	71.1 (1496)	81.5 (678)	64.3 (818)	<0.005
Symptoms of COVID-19 infection include runny nose, headache, cough, sore throat, fever, shortness of breath and general feeling of being unwell.	91.6 (1927)	94.2 (784)	89.9 (1143)	<0.005
Complications of coronavirus affect all individuals equally regardless of their health status. *	83.8 (1764)	76.7 (638)	88.5 (1126)	<0.005
Death from coronavirus infection is a very common complication.*	44.2 (929)	19.2 (160)	60.5 (769)	<0.005
People with cardiopulmonary disease, people with weakened immune systems, infants, pregnant ladies and older adults are at increased risk of infection.	89.4 (1881)	84.9 (706)	92.4 (1175)	<0.005
**Mode of transmission** † **[mean score ± SD]**	**[3.2 ± 0.8]**	**[3.2 ± 0.7]**	**[3.3 ± 0.8]**	**0.037**
Respiratory droplets by coughing and sneezing, close personal contact, such as touching or shaking hands.	83.3 (1752)	92.9 (773)	77.0 (979)	<0.005
Sexual contact. *	82.9 (1745)	83.1 (691)	82.9 (1054)	0.909
Blood transfusion. *	75.4 (1586)	71.5 (595)	77.9 (991)	0.001
Touching an object or surface with the virus on it, then touching your mouth, nose, or eyes before washing your hands.	81.7 (1720)	71.6 (596)	88.4 (1124)	<0.005
**Prevention of transmission and treatment**† **[mean score ± SD]**	**[7.4±1.1]**	**[6.9 ± 1.1]**	**[7.6 ± 1.1]**	**<0.005**
Influenza vaccines may protect you against human coronavirus infection.*	60.2 (1266)	55.2 (459)	63.4 (807)	<0.005
Currently, there is specific treatment for illnesses caused by the new coronaviruses.*	64.6 (1359)	65.1 (542)	64.2 (817)	0.682
Frequently clean hands by using alcohol-based hand rub or soap and water.	97.3 (2047)	95.0 (790)	98.8 (1257)	<0.005
Avoid touching your eyes, nose, or mouth with unwashed hands.	96.2 (2023)	92.9 (773)	98.3 (1250)	<0.005
When coughing and sneezing cover mouth and nose with flexed elbow or tissue—throw tissue away immediately and wash hands.	97.1 (2044)	95.9 (798)	98.0 (1246)	0.007
Avoid close contact with people who are sick	98.6 (2074)	98.4 (819)	98.7 (1255)	0.092
Avoid spitting in public.	95.2 (2003)	92.3 (768)	97.1 (1235)	<0.005
If you have fever, cough and difficulty breathing seek medical care early and share previous travel history with your health care provider. #	98.2 (2066)	97.8 (814)	98.4 (1252)	0.136
Use a facemask every day to protect yourself.*¥	18.3 (385)	2.6 (22)	28.5 (363)	<0.005
**Knowledge score [mean score ± SD]**	**[15.9 ± 2.2]**	**[15.2 ± 2.2]**	**[16.3 ± 2.1]**	**<0.005**

* The correct answer for the question is “NO”.

^†^ Participants were asked to choose answers as many as applies.

^#^ At the early days of the pandemic, part of the diagnosis relied on the travel history of the patient, specifically if returning from epicenters of the pandemic.

^¥^At the early days of the pandemic, WHO, CDC and MOH recommendations were to use facemasks by patients only and not the public, to spare the facemasks for the medical workers.

Regarding COVID-19 transmission, a major gap of knowledge regarding routes of COVID-19 transmission was identified where only 2.3% (n = 49) of the population correctly recognized all the routes of COVID-19 transmission.

Considering COVID-19 preventative measures, more than half of the participants were able to identify the main preventative measures. However, only 18.3% (n = 385) of them knew that a facemask should not be used daily as a preventative measure for COVID-19 transmission (which was a recommendation by WHO and MOH at the early days of the pandemic).

In general, the mean knowledge scores of participants improved after case reporting (when compared with those exhibited before case reporting) in the three areas studied: Definition, signs and symptoms, risk groups and complications (mean score 5.5 ±1.2 vs 5.1 ±1.2, p<0.005); Mode of transmission (mean score 3.3 ± 0.8 vs 3.2 ± 0.7, p = 0.037) and Prevention of transmission and treatment (mean score 7.6 ± 1.1 vs 6.9 ± 1.1, p<0.005). Accordingly, the total knowledge score was improved from 15.2 ± 2.2 before case reporting to 16.3 ± 2.1 after case reporting (p value <0.001, independent sample t-test) (Table 2). The detailed frequencies for individual questions are presented in Table 2.

### Sources of information about COVID-19 infection

The most common source of participants’ information about COVID-19 was social media, such as Facebook, twitter or others (64.3%, *n* = 1353), followed by internet searching such as Google (59.4%, n = 1249), and television (49·7%, n = 1046). A much lower proportion of respondents relied on newspapers (37.3%, n = 784), friends (29.3%, n = 616), brochures (20.2%, *n* = 426) or physicians’ offices (6.6%, n = 138) to get information. A significantly higher knowledge score (*p*-value < 0.001) was associated with using internet search (score = 16.4) as sources of information (*P*-value ˂ 0.001; independent sample t-test), while other sources were not associated with significantly higher knowledge score.

### Participants’ attitudes towards COVID-19 and its preventative measures

Most participants had positive attitudes regarding their role in preventing COVID-19. In particular, 67.2% of participants thought that they could protect themselves against COVID-19 and 88.7% of them thought that following advised preventative measures would be effective. However, more than 50% of participants didn’t trust the Jordanian Ministry of Health’s (MOH) approach to confronting COVID-19 (57.6%, n = 1211) or the information provided by Governmental authorities about the exact number of cases (66.1%, n = 2091). Similarly, more than half of participants believed that the occurrence of coronavirus was related to international tension and trade wars (57.9%, n = 1219) and 49.7% (n = 1046) of participants believed that the recent coronavirus was created in a laboratory and was not naturally occurring. On the other hand, most participants were either unsure or didn’t believe that herbal remedies (71.7%, n = 1508) or antibiotics (85.9%, n = 1808) were effective in treating or preventing COVID-19.

Interestingly, many of the participants’ attitudes had changed after reporting the first Jordanian case of COVID-19 (2^nd^ March 2020). Most importantly, the percentage of participants who believe that COVID-19 was a serious and life threating infection dropped significantly from 81.6% to 52.6% (p value < 0.001; Table 3). On the other hand, the proportion of participants who trusted MOH in confronting COVID-19 increased significantly from 35.9% to 46.7% (p value<0.001). Similarly, the percentage of participants who thought that they could protect themselves against infection with COVID-19 (59.3% vs.72.3%, p value <0.005) and those who thought that treatment approaches were effective also increased significantly (from 31.0% to 42.7%, p value = 0.009).

**Table 3:** Attitudes of participants towards COVID-19 before and after reporting cases in Jordan 2^nd^ March 2020.

Participants attitudes	Overall participants (n = 2104)	Before (n = 832)	After (n = 1272)	p-value*
	% (n)			
Think that COVID-19 is a serious and life threating infection.	64.9 (1366)	81.6 (679)	52.6 (669)	*<0*.*001*
Think that you can protect yourself against infection with COVID-19.	67.2 (1413)	59.3 (493)	72.3 (920)	*<0*.*001*
Think that following the preventative measures are effective.	88.7 (1866)	86.2 (717)	90.3 (1149)	*0*.*009*
Think that treatment approaches are effective	38.1 (801)	31.0 (258)	42.7 (543)	*<0*.*001*
Trust MOH in Jordan in confronting COVID-19	42.4 (893)	35.9 (299)	46.7 (594)	*<0*.*001*
Think that governmental authorities provide individuals with trusted information about coronavirus (for example information related to the number of infected individuals in Jordan)	33.9 (713)	32.0 (266)	35.1 (447)	0.28
Will buy the vaccine if it was provided for COVID-19.	57.5 (1210)	56.0 (466)	58.5 (744)	0.343
Will accept to take the vaccine if it was offered for free by the government.	66·1 (1391)	63·3 (527)	67·9 (864)	0.084
Think that the recent coronavirus was created in a lab and not naturally occurring	49.7 (1046)	46.9 (390)	51.7 (658)	0.105
Think that the time of coronavirus appearance is related to international tension and trade wars	57.9 (1219)	57.1 (475)	58.5 (744)	0.733
Think that herbal remedies (for example star anise, garlic etc) are effective in treating or preventing coronavirus infection	28.3 (596)	26.1 (217)	29.8 (379)	0.167
Think that antibiotics are effective in treating or preventing coronavirus infection	14.1 (296)	14.2 (118)	14.0 (178)	0.256
If you or one of your family members developed fever, what will you do				
Visit emergency department immediately	34.2 (719)	34.7 (289)	33.8 (430)	*0*.*026*
Visit a physician immediately	4.8 (101)	6.4 (53)	3.8 (48)	
Take an antipyretic	4.8 (100)	5.5 (46)	4.3 (54)	
Keep monitoring and if symptoms worsen, visit a physician or emergency department	54.8 (1153)	52.0 (433)	56.6 (720)	
No action	1.5 (31)	1.3 (11)	1.6 (20)	

* p values were calculated by Chi-Square test

### Participants’ practices to prevent transmission of COVID-19

The mean practice score of all the participants was 3.95 ± 1.7 (out of the 6 practice questions) with 66.7% (*n* = 1403) of the participants having a score equal to or higher than 4 and consequently were considered as having adequate practices to prevent spread of COVID-19. Respondents’ practices are presented in Table 4. Participants’ practices to prevent transmission of COVID-19 in terms of avoiding hand shaking, hugging, kissing and crowded areas, and using disinfectants were appropriate in more than 60% of participants. Interestingly, all these practices have increased significantly after reporting the first case of COVID-19. On the other hand, the proportion of participants wearing facemasks and avoiding the purchase of Chinese goods have decreased significantly from 45.9% to 34.8% and from 54.8% to 42.2% respectively (p value < 0.001; Table 4). Overall, the observed practice score improved significantly when compared between respondents before case reporting and after case reporting (3.65 ± 1.9 vs. 4.2 ± 1.4 respectively, p value < 0.001, independent sample t-test; Table 4).

**Table 4:** Participants’ practices that were changed after reporting cases of COVID-19 in Jordan, 2^nd^ March 2020.

Question	Total participants	Before	After	P value
	**% (n)**			
Avoid shaking hand with others	64.6 (1353)	49.6 (410)	74.5 (943)	<0.001
Avoid hugging/kissing others	80.0 (1676)	67.4 (557)	88.2 (1119)	<0.001
Avoid crowded areas	84.4 (1764)	77.8 (644)	88.7 (1120)	<0.001
Wear facemask when getting outside *	39.2 (822)	45.9 (380)	34.8 (442)	<0.001
Increase in the use of disinfectants like Dettol^®^, Hypex^®^, HiGeen^®^ to clean and disinfect objects and surfaces	81.9 (1712)	71.4 (590)	88.7 (1122)	<0.001
Avoid purchasing goods of Chinese origin† [26, 27]	47.1 (986)	54.8 (452)	42.2 (534)	<0.001
**Practice score [mean score ± SD]**	**[3.95 ± 1.7]**	**[3.65 ± 1.9]**	**[4.2 ± 1.4]**	**<0.001**

* At the early days of the study, WHO, CDC and MOH recommendations were to use facemasks by patients only, to spare the facemask for the medical worker.

^**†**^ At the time of commencing the study, China was the epicenter of the disease.

### Factors affecting participants’ knowledge and practices towards COVID-19 and its preventative measures

Factors affecting participants’ knowledge and practice towards preventing COVID-19 were determined by bivariate analysis. Overall participants’ knowledge and practice scores were associated with age, educational level and education in health or medical field. In addition, participants’ knowledge score was associated with gender and work in the medical field. Participants’ practice score was also associated with marital status. For each sociodemographic characteristic, the calculated knowledge score (Table 5) or practice score (Table 6) was improved after case reporting when compared to the value before case reporting. Detailed results are shown in Tables 5 and 6.

**Table 5:** Factors affecting participants’ knowledge towards COVID-19 and its preventative measures.

	Overall participants’ knowledge scores towards coronavirus and its preventative measures #	P value within group	Participants’ knowledge scores before documentation of the first COVID case#	Participants’ knowledge scores after documentation of the first COVID case#	P value* between groups
	Mean ± SD	P value	Mean ± SD	Mean ± SD	P value
**Age group**					
**18–25**	**16.0±2.3**	***0*.*035***^†^	***15*.*1* ±2.4**	***16*.*1* ±2.2**	***<0*.*005***
**26–35**	**16.3±2.3**		**15.5 ±2.1**	**16.4 ±2.2**	**<0.005**
**36–45**	**15.9±2.0**		**15.1 ±2.0**	**16.4 ±1.9**	**<0.005**
**46–55**	**15.9±2.2**		**15.0 ±2.2**	**16.4 ±2.0**	**<0.005**
**>55**	**16.0±2.3**		***15*.*2* ±2.3**	***16*.*2* ±2.3**	***0*.*018***
**Gender**					
**Male**	**15.8±2.5**	*0*.003*	***14*.*9*±2.7**	***16*.*0* ±2.4**	***<0*.*005***
**Female**	**16.1±2.1**		**15.3 ±2.0**	**16.4 ±2.0**	**<0.005**
**Marital status**					
**Married**	**16.0±2.1**	**0.683**^†^	**15.2 ±2.1**	**16.4 ±2.1**	**<0.005**
**Single**	**16.1±2.3**		**15.2 ±2.1**	**16.4 ±2.1**	**<0.005**
**Others**	**16.1±2.1**		**15.7 ±2.2**	**16.1 ±2.1**	**0.444**
**Educational level**					
**Post graduate**	**16.2±2.4**	***<0*.*001***^†^	***15*.*3* ±2.4**	***16*.*8* ±2.2**	***<0*.*005***
**University level**	**16.2±2.2**		**15.4 ±2.2**	**16.5 ±2.1**	**<0.005**
**Diploma**	**15.4±2.0**		**14.7 ±1.9**	**15.5 ±2.0**	**0.008**
**12 years or less**	**15.3±2.1**		**14.2 ±2.1**	**15.4 ±2.2**	**0.001**
**Education in health or medical field**					
**Yes**	**16.8±1.8**	*<0*.001*	***6*.*0* ±1.8**	***17*.*1* ±1.8**	***<0*.*005***
**No**	**15.5±2.3**		**14.5 ±2.4**	**15.8 ±2.1**	**<0.005**
**Work in medical field**					
**Yes**	**16.8±1.9**	*<0*.001*	***14*.*5* ±2.4**	***15*.*8* ±2.2**	***<0*.*005***
**No**	**15.7±2.3**		**14.8 ±2.2**	**16.0 ±2.2**	**<0.005**

*p values were calculated using independent sample t test,

^†^ p values were calculated by one way ANOVA,

^#^ the maximum value of this score is 20.

**Table 6:** Factors affecting participants’ practices towards COVID-19 and its preventative measures.

	Overall participants practice scores towards coronavirus and its preventative measures #	P value within group	Participants’ practice scores before documentation of the first COVID case#	Participants’ practice scores after documentation of the first COVID case#	P value* between groups
	Mean ± SD		Mean ± SD	Mean ± SD	
**Age group**					
18–25	**3.8±1.8**	**<0.001**^†^	**3.4 ±2.0**	**4.0 ±1.6**	**<0.005**
26–35	**3.9±1.7**		**3.4 ±2.1**	**4.1 ±1.4**	**<0.005**
36–45	**4.1±1.6**		**4.0 ±1.8**	**4.3 ±1.4**	**0.083**
46–55	**4.1±1.7**		**3.9 ±1.9**	**4.3 ±1.4**	**0.062**
>55	**4.5±1.4**		**4.5 ±1.6**	**4.4 ±1.3**	**0.686**
**Gender**					
Male	**3.9±1.7**	0.345*	**3.6 ±2.0**	**4.1 ±1.5**	**0.002**
Female	**4.0±1.7**		**3.7 ±2.0**	**4.2 ±1.4**	**<0.005**
**Marital status**					
Married	**4.1±1.7**	**0.001**^†^	**3.9 ± 1.9**	**4.2 ± 1.4**	**0.003**
Single	**3.8±1.7**		**3.3 ± 2.1**	**4.1 ± 1.5**	**<0.005**
Others	**4.1±1.5**		**3.7 ± 1.7**	**4.4 ± 1.2**	**0.117**
**Educational level**					
Post graduate	**3.9±1.7**	**<0.001**^†^	***3*.*7* ±1.9**	***4*.*2* ±1.3**	***0*.*005***
University level	**3.9±1.7**		**3.6 ±2.0**	**4.0 ±1.5**	**<0.005**
Diploma	**4.4±1.5**		**4.3 ±1.8**	**4.5 ±1.3**	**0.276**
12 years or less	**4.3±1.7**		**3.6 ± 1.9**	**4.5 ±1.5**	**0.003**
**Education in health or medical field**					
Yes	**3.8±1.7**	0.001*	**3.5 ±1.9**	**4.1 ±1.3**	**<0.005**
No	**4.1±1.7**		**3.8 ±2.0**	**4.2 ±1.5**	**<0.005**
**Work in medical field**					
Yes	**3.9±1.6**	0.110*	**3.5 ±1.9**	**4.1 ±1.3**	**<0.005**
No	**3.9±1.7**		**3.7 ±2.0**	**4.2 ±1.5**	**<0.005**

*p values were calculated using independent sample t test,

^†^ p values were calculated by one way ANOVA,

^#^ the maximum value of this score is 6.

## Discussion

Since the WHO declaration of COVID-19 as a public health emergency on 30^th^ January 2020, health authorities around the world, led by WHO, have initiated huge campaigns to increase the awareness of the people toward the disease and to disseminate the appropriate practices to prevent its transmission [4]. As a member of these authorities, the MOH in Jordan started similar campaigns relying on different forms of media. The first case in Jordan was reported on 2^nd^ March 2020 for a citizen returning from Italy [28]. Our study commenced by distributing the questionnaires on 17^th^ February before the reporting of the first case of COVID-19 in Jordan, and the questionnaire process ended on 19^th^ March. By that time, the measures taken by the country included closure of schools and universities, quarantining thousands of incoming air passengers in hotels, and prohibiting any kind of social gathering (including the closure of all mosques and churches). During that period, the number of cases reported increased to 52 without fatality. This gave our team an opportunity to compare the change in knowledge, attitudes and practices of Jordanians towards COVID-19 before and after the reporting cases of COVID-19 illness.

The sample population of this study (2104 participants) was largely well educated with females predominating. The observed skew in the sample toward females and well educated participants has also been seen in previous studies in Jordan [29–32]. In addition, the vast majority of participants were below the age of 55 years, which is representative of the generally younger population of Jordan, where at the end of 2019 the estimated proportion of population below 55 years was around 92% [33]. The overall knowledge of the respondents was generally adequate (total knowledge score 15.9 out of 20). They exhibited excellent knowledge of the organs targeted by the virus, its nature as a zoonotic disease, and its signs and symptoms. However, the respondents had an exaggerated idea about the expected death rate of the disease where more than 55% considered death to be a very common outcome. This idea could have been drawn in their minds due to the effect of media focus on death rates and novel cases, rather than recovered cases [34, 35], thus, giving the audience or readers the impression that this disease is highly fatal.

The respondents were well aware of the most common routes of the disease transmission, such as close contact, respiratory droplets or touching contaminated surfaces.

The vast majority of participants (> 90%) had an excellent knowledge about the required measures to prevent the disease. However, the more scientific questions related to the role of influenza vaccine in protecting from COVID-19 or the one related to use of a specific drug to treat the illness were correctly answered by only around two thirds the participants. The participants’ knowledge was gained through multiple sources of information. The electronic sources such as social media (64%) and internet (59%) were the top sources followed by television (50%). These results suggest that in this era, where electronic media sources are predominate at the expense of more traditional media sources, health authorities should focus their health-related campaigns on electronic media and they should consider adding social media platforms to their public communication tools, in order to reach the vast majority of population [36–39]. It is noteworthy that people who obtained their information from internet searches had significantly higher knowledge scores than those using other sources. This is probably due to the fact that people who were keen to get more information about the disease actively sought this information from the internet. However, those relying on social media, such as Facebook, twitter and WhatsApp may have received inaccurate information since social media platforms often lack fact-checking and editorial control [40, 41].

When investigating the knowledge of the participants the major factors were; the educational level, education background, and the field of work (P value within group <0.005 for each) (Table 5). This was anticipated and is in line with previous studies that compare the knowledge of the general population about a specific health-related issue with those of higher levels of education or with a health-related backgrounds [42, 43]. The latter would have much better knowledge. Comparing the mean knowledge scores of the respondents before and after case reporting (Table 2), showed an improvement in the respondents’ knowledge after case reporting (P value between groups <0.05, independent sample t-test). We are aware that differences in the population characteristics before and after case reporting might contribute to observed changes in the knowledge score. However, statistical analysis of the factors affecting participants’ knowledge (Table 5) exhibits that the observed improvement in the knowledge was significant in all the classes of each characteristic (age, gender, marital status, etc, P value between groups <0.05), indicating a palpable effect of case reporting on the participants’ knowledge. Apparently, people become more interested in learning about a disease when it is seen to be proximal to their vicinity as has been seen in other similar situations [18]. This suggests the importance of pursuing health campaigns and associated recommendations when case reporting of any outbreak or epidemic commences in a country, as the public seems to become more receptive to that additional information.

A significant change in the attitudes of participants in many aspects was noticed after reporting cases of COVID-19 illnesses in Jordan. Unexpectedly, the participants seemed to become more optimistic and comfortable after the reporting of cases of COVID-19. This optimism appeared in their perception to the severity of disease as a life threatening one, the effectiveness of preventative measures, their ability to protect themselves, the effectiveness of the treatment approaches and the ability of MOH to confront the disease. Although this is initially puzzling, it can be explained by the unique situation in Jordan. Shortly after first case reporting in Jordan, there was a sense of panic among the population. In order to calm the public and fight the rumors, the first COVID-19 patient was interviewed in hospital where he was isolated, by different national and regional TV and radio channels [44]. The patient tried to comfort the people about his situation, since he only suffered from mild respiratory illness. Being a traveler returning back from Italy, which was hard hit by the pandemic at that time, he compared the looser measures taken by Italian authorities to contain the disease with the tougher measures taken in Jordan, and he praised the latter. The same thing happened with the second case who only suffered from mild illness. The situation of both patients was of great public interest until they were discharged from hospital. Apparently, the message perceived by the public from both patients was that the disease is mild and can be prevented, and that the measures taken by the authorities are effective. Our study suggests that the Jordanian Government’s open approach with the public about the disease, its spread, and the necessity for stringent control measures enhanced the optimistic attitudes of the participants towards the disease and its management after case reporting in the country.

On the other hand, the attitudes of the participants in other areas remained the same before and after reporting cases in Jordan. More than half the respondents indicated their willingness to buy the vaccine (most pharmaceuticals are paid for by the patient in Jordan) and the number increased a little more if the vaccine was offered free of charge. These figures are relatively high compared to the general attitudes of Jordanians towards vaccinations other than the mandatory ones at childhood [42, 45]. This improved attitude could be related to the worldwide worries about this disease, which have made the Jordanians more willing to protect themselves against coronavirus. As for the emergence of this virus, almost half of the participants believed that the virus was created in a laboratory and more than half believed that its emergence was the result of political reason linked to international tension or trade war. This conspiracy theory is not limited to Jordanians, and has been reported worldwide. Some Americans accused China of bioengineering the virus as a bioweapon and conversely, some Chinese have accused the American military of introducing it into China. Nevertheless, these theories have been refuted in the media and by scientific studies [46–49].

The practices of the participants in order to prevent the transmission of the virus were found to be adequate for more than two thirds of the respondents. However, there was a remarkable improvement (p < 0.001) in the participants’ practices after the reporting of cases of the disease in Jordan. The practices evaluated included; avoidance of shaking hands, avoidance of kissing, avoidance of crowded places, and an increase in the use of disinfectants (Table 4). This improvement in practices’ scores was anticipated because the public was observing the disease having increased proximity to their areas, thus they were more willing to engage with the recommendations of the MOH regarding COVID-19. The improvement in practices’ scores seen among the population after case reporting compared to those before case reporting cannot be attributed solely to differences in sample population, because the increase in practice score was obvious in all the population characteristics studied (Table 6). After reporting cases in Jordan, there was a significant decrease in the number of participants using facemasks when going outside. This contradicts the behavior of Chinese people during the epidemic in their region, where 98% of a study population indicated wearing facemasks when leaving homes [50]. Nevertheless in this respect, the practices of Jordanian respondents exhibited better adherence to the instructions of MOH at the time. In the early days of the pandemic WHO and MOH instructions to the general public were not to use facemasks, as it was feared this would lead to shortage of masks for essential health and care workers. Many other factors affected the overall practices of the respondents. Participants with ages of more than 55 years had statistically better practices than those in the lower age groups (18-25yr and 26-35yr; P value within group <0.005). This implies that older respondents are more cautious about their health than younger participants due to WHO and MOH warnings that people above the age of 60 years were at higher risk of developing severe complications than those of younger ages [51]. These findings suggest that the public is willing to implement the recommendations of the health authorities when continuously and firmly directed to do so.

Surprisingly, the negative factors affecting the overall practices of the participants towards the prevention of transmission are the education level and having a health-related education. These participants scored lower than their counterparts in terms of the best practices (Table 6). This finding, albeit odd is not uncommon. In a study among health care workers in Saudi Arabia to evaluate their KAP about Middle East Respiratory Syndrome (MERS), physicians, pharmacists, nurses and technicians showed low to average scores on practices, but better knowledge and attitude scores [52]. We speculate that having a better education, particularly in a health-related field, provides the participants with confidence that they are aware of the risks and know better how to protect themselves.

Another disappointing finding is that the overall participants practice score of those working in the health field is the same as those working outside this field (3.9 for each, Table 6). This finding should be alarming to the health authorities and health care sector, since these workers will be in the front line in combating the epidemic. Being lenient in applying the strictest measures and practices in protecting themselves and preventing the spread of infection could lead to a disaster. Therefore, the health sector should immediately intensify education and extensive training and setting guidelines for the proper practices among healthcare workers of different specialties.

### Limitations of the study

The findings of the study should be interpreted with the following limitations in mind:

- Sampling of the study was made through social media, What’s App in particular, which could pose some bias to the study where underprivileged people or those having problems in using electronic devices may not be able to participate, thus, will be under-represented in the sample.
- Females and people with education level of more than 12 years were over-represented in the sample studied when compared to the general Jordanian population.
- Like any other self-reported study, responses (mainly of attitudes and practices) could have been reported based on social desirability, not the actual situation of the participants.

## Conclusions

The design and timing of this study could not have been more fortuitous, in that it allowed the authors to rapidly adapt and expand a preliminary study into a fully operational before-and-after study of KAP towards COVID-19. The findings of this study are necessarily complex, but generally show that the population reacts well to open and honest governmental advice about pandemics and can rapidly adopt safe practices in response to appropriate advice. The international relevance of this work is obvious, in that Jordan achieved one of the lowest rates (per million population) of COVID-19 infection ranking 186 and 177 out of 213 in the number of cases and related fatality respectively, in the world (https://www.worldometers.info/coronavirus/#countries; 14/08/20).

## References

[1] Lai CC, Shih TP, Ko WC, Tang HJ, Hsueh PR. Severe acute respiratory syndrome coronavirus 2 (SARS-CoV-2) and coronavirus disease-2019 (COVID-19): The epidemic and the challenges. Int J Antimicrob Agents. Elsevier B.V.; 2020;55 10.1016/j.ijantimicag.2020.105924 32081636 PMC7127800

[2] WHO. Coronavirus. In: WHO [Internet]. 2020 [cited 1 Apr 2020]. https://www.who.int/health-topics/coronavirus#tab=tab_1

[3] CDC. Coronavirus Disease 2019 (COVID-19), Symptoms of Coronavirus. In: Centers for Disease Control and Prevention [Internet]. 2020 [cited 7 Apr 2020]. https://www.cdc.gov/coronavirus/2019-ncov/symptoms-testing/symptoms.html?CDC_AA_refVal=https%3A%2F%2Fwww.cdc.gov%2Fcoronavirus%2F2019-ncov%2Fabout%2Fsymptoms.html

[4] WHO. Events as they happen-Rolling updates on coronavirus disease (COVID-19) [Internet]. 2020 [cited 30 Mar 2020]. https://www.who.int/emergencies/diseases/novel-coronavirus-2019/events-as-they-happen

[5] Li Q, Guan X, Wu P, Wang X, Zhou L, Tong Y, et al Early Transmission Dynamics in Wuhan, China, of Novel Coronavirus–Infected Pneumonia. N Engl J Med. Massachusetts Medical Society; 2020; 10.1056/nejmoa2001316 31995857 PMC7121484

[6] Cheng ZJ, Shan J. 2019 Novel coronavirus: where we are and what we know. Infection. Springer; 2020;48: 155–163. 10.1007/s15010-020-01401-y 32072569 PMC7095345

[7] McCreary EK, Pogue JM. Coronavirus disease 2019 treatment: A review of early and emerging options [Internet]. Open Forum Infectious Diseases. Oxford University Press; 2020 10.1093/ofid/ofaa105 32284951 PMC7144823

[8] WHO. Draft landscape of COVID-19 candidate vaccines [Internet]. Jul 2020 [cited 11 Aug 2020]. https://www.who.int/publications/m/item/draft-landscape-of-covid-19-candidate-vaccines

[9] WHO. Modes of transmission of virus causing COVID-19: implications for IPC precaution recommendations [Internet]. Mar 2020 [cited 11 Aug 2020]. https://www.who.int/news-room/commentaries/detail/modes-of-transmission-of-virus-causing-covid-19-implications-for-ipc-precaution-recommendations

[10] WHO. Q&As on COVID-19 for older people [Internet]. 2020 [cited 11 Aug 2020]. https://bit.ly/2Y5M8tR.

[11] The World Bank. Refugee population by country or territory of asylum—| Data [Internet]. 2018 [cited 7 Apr 2020]. https://data.worldbank.org/indicator/SM.POP.REFG?locations=JO%2C&view=map

[12] Nazer LH, Tuffaha H. Health care and pharmacy practice in Jordan. Can J Hosp Pharm. Canadian Society of Hospital Pharmacists; 2017;70: 150–155. 10.4212/cjhp.v70i2.1649 28487583 PMC5407425

[13] Higher Health Council & World Health Organization. Jordan National Health Sector Strategy 2015–2019. In: USAID, Jordan [Internet]. 2015 [cited 8 Apr 2020]. https://jordankmportal.com/resources/jordan-national-health-sector-strategy-2015-2019

[14] Roya News. Government suspends educational system in a bouquet of coronavirus preventive measures [Internet]. 14 Mar 2020 [cited 8 Apr 2020]. https://en.royanews.tv/news/20223/Government-suspends-educational-system-in-a-bouquet-of-coronavirus-preventive-measures

[15] Roya News. Government announces 10 decisions to fight against coronavirus. In: Roya News [Internet]. 10 Mar 2020 [cited 8 Apr 2020]. https://en.royanews.tv/news/20201/Government_announces_10_decisi

[16] Al Sharif O. Under nationwide curfew, Jordanians now ponder economic cost of coronavirus. In: Al-Monitor, The Pulse of the Middle East [Internet]. 23 Mar 2020 [cited 1 Apr 2020]. https://www.al-monitor.com/pulse/originals/2020/03/jordan-lockdown-economy-measures-coronavirus.html

[17] WHO. Coronavirus disease (COVID-19) advice for the public. In: WHO [Internet]. 2020 [cited 1 Apr 2020]. https://www.who.int/emergencies/diseases/novel-coronavirus-2019/advice-for-public

[18] Al-Hazmi A, Gosadi I, Somily A, Alsubaie S, Bin Saeed A. Knowledge, attitude and practice of secondary schools and university students toward Middle East Respiratory Syndrome epidemic in Saudi Arabia: A cross-sectional study. Saudi J Biol Sci. Elsevier B.V.; 2018;25: 572–577. 10.1016/j.sjbs.2016.01.032 29686521 PMC5910645

[19] CDC. Coronavirus Disease 2019 (COVID-19)-How Coronavirus Spreads. In: Centers for Disease Control and Prevention [Internet]. 2020 [cited 9 Apr 2020]. https://www.cdc.gov/coronavirus/2019-ncov/prevent-getting-sick/how-covid-spreads.html?CDC_AA_refVal=https%3A%2F%2Fwww.cdc.gov%2Fcoronavirus%2F2019-ncov%2Fprepare%2Ftransmission.html

[20] CDC. Coronavirus Disease 2019 (COVID-19)-How to Protect Yourself & Others. In: Centers for Disease Control and Prevention [Internet]. 2020 [cited 9 Apr 2020]. https://www.cdc.gov/coronavirus/2019-ncov/prevent-getting-sick/prevention.html?CDC_AA_refVal=https%3A%2F%2Fwww.cdc.gov%2Fcoronavirus%2F2019-ncov%2Fprepare%2Fprevention.html

[21] Azlan AA, Hamzah MR, Sern TJ, Ayub SH, Mohamad E. Public knowledge, attitudes and practices towards COVID-19: A cross-sectional study in Malaysia. Tu W-J, editor. PLoS One. Public Library of Science; 2020;15: e0233668 10.1371/journal.pone.0233668 32437434 PMC7241824

[22] Reuben RC, Danladi MMA, Saleh DA, Ejembi PE. Knowledge, Attitudes and Practices Towards COVID-19: An Epidemiological Survey in North-Central Nigeria. J Community Health. Springer; 2020; 1–14. 10.1007/s10900-020-00881-1 32638198 PMC7338341

[23] Parker C, Scott S, Geddes A. Snowball Sampling In: Atkinson P, Delamont S, Cernat A, Sakshaug J, Williams R, editors. SAGE Research Methods Foundations. SAGE Publications Ltd; 2020.

[24] Lino M, Di Giuseppe G, Albano L, Angelillo IF. Parental knowledg attitudes and behaviours towards influenza A/H1N1 in Italy. Eur J Public Health. Oxford Academic; 2012;22: 568–572. 10.1093/eurpub/ckr115 21873277

[25] van Griethuijsen RALF, van Eijck MW, Haste H, den Brok PJ, Skinner NC, Mansour N, et al Global patterns in students’ views of science and interest in science. Res Sci Educ. Kluwer Academic Publishers; 2015;45: 581–603. 10.1007/s11165-014-9438-6

[26] Tass RNA. WHO says people receiving packages from China not at risk of contracting coronavirus—Society & Culture—TASS [Internet]. Feb 2020 [cited 11 Aug 2020]. https://tass.com/society/1115385

[27] Kreiger L. Can I get coronavirus from a package delivered from China?–Daily News. In: Los Angeles Daily News [Internet]. 2020 [cited 11 Aug 2020]. https://www.dailynews.com/2020/02/26/can-i-get-coronavirus-from-a-package-being-delivered-from-china/

[28] WHO. Coronavirus disease 2019 (COVID-19) Situation Report– 43 [Internet]. 3 Mar 2020 [cited 30 Mar 2020]. https://www.who.int/docs/default-source/coronaviruse/situation-reports/20200303-sitrep-43-covid-19.pdf?sfvrsn=76e425ed_2

[29] Sharaydih R, Abuloha S, Wazaify M. Promotion of appropriate knowledge and attitude towards medicines among schoolchildren in Jordan: the role of teachers. Int J Pharm Pract. Wiley-Blackwell Publishing Ltd; 2020;28: 84–91. 10.1111/ijpp.12582 31573122

[30] Olaimat AN, Aolymat I, Shahbaz HM, Holley RA. Knowledge and Information Sources About COVID-19 Among University Students in Jordan: A Cross-Sectional Study. Front Public Heal. Frontiers Media S.A.; 2020;8 10.3389/fpubh.2020.00254 32574314 PMC7274134

[31] Shehadeh M, Suaifan G, Darwish RM, Wazaify M, Zaru L, Alja’fari S. Knowledge, attitudes and behavior regarding antibiotics use and misuse among adults in the community of Jordan. A pilot study. Saudi Pharm J. 2012;20: 125–133. 10.1016/j.jsps.2011.11.005 23960783 PMC3744980

[32] Abu-Rish EY, Elayeh ER, Mousa LA, Butanji YK, Albsoul-Younes AM. Knowledge, awareness and practices towards seasonal influenza and its vaccine: Implications for future vaccination campaigns in Jordan. Fam Pract. Oxford University Press; 2016;33: 690–697. 10.1093/fampra/cmw086 27567011 PMC7188315

[33] Department of Statistics-Jordan Government. Table 2: Estimated Population of the Kingdom by Sex, Age Group and Sex Ratio 2004–2019. PxWeb. In: Interactive database [Internet]. 2020 [cited 11 Aug 2020]. http://jorinfo.dos.gov.jo/Databank/pxweb/en/Demographi_Statistics/Demographi_Statistics/Table2.px/

[34] Chris Mooney, Sarah Kaplan and, Min Joo Kim. Why coronavirus is killing far more men than women—The Washington Post. The Washington Post. 19 Mar 2020. https://www.washingtonpost.com/climate-environment/2020/03/19/coronavirus-kills-more-men-than-women/. Accessed 30 Mar 2020.

[35] The Jordan Times. More COVID-19 cases, deaths reported in rest of world than in China—WHO. In: The Jordan Times [Internet]. 16 Mar 2020 [cited 8 Apr 2020]. http://jordantimes.com/news/world/more-covid-19-cases-deaths-reported-rest-world-china-—-who

[36] Alhaddad MS. The use of social media among Saudi residents for medicines related information. Saudi Pharm J. Elsevier B.V.; 2018;26: 1106–1111. 10.1016/j.jsps.2018.05.021 30510470 PMC6257910

[37] Zucco R, Lavano F, Anfosso R, Bianco A, Pileggi C, Pavia M. Internet and social media use for antibiotic-related information seeking: Findings from a survey among adult population in Italy. Int J Med Inform. Elsevier Ireland Ltd; 2018;111: 131–139. 10.1016/j.ijmedinf.2017.12.005 29425624

[38] Thurman N, Myllylahti M. Taking the paper out of news. Journal Stud. Taylor & Francis Group; 2009;10: 691–708. 10.1080/14616700902812959

[39] Bawazir A, Al-Mazroo E, Jradi H, Ahmed A, Badri M. MERS-CoV infection: Mind the public knowledge gap. J Infect Public Health. Elsevier Ltd; 2018;11: 89–93. 10.1016/j.jiph.2017.05.003 28647126 PMC7102865

[40] Loeb S, Taylor J, Borin JF, Mihalcea R, Perez-Rosas V, Byrne N, et al Fake News: Spread of Misinformation about Urological Conditions on Social Media. Eur Urol Focus. Elsevier B.V.; 2019; 10.1016/j.euf.2019.11.011 31874796

[41] Brady JT, Kelly ME, Stein SL. The Trump Effect: With No Peer Review, How Do We Know What to Really Believe on Social Media? Clin Colon Rectal Surg. Thieme Medical Publishers, Inc.; 2017;30: 270–276. 10.1055/s-0037-1604256 28924401 PMC5595538

[42] Assaf AM, Hammad EA, Haddadin RN. Influenza Vaccination Coverage Rates, Knowledge, Attitudes, and Beliefs in Jordan: A Comprehensive Study. Viral Immunol. Mary Ann Liebert Inc.; 2016;29: 516–525. 10.1089/vim.2015.0135 27509083

[43] Brouard C, Gautier A, Saboni L, Jestin C, Semaille C, Beltzer N. Hepatitis B knowledge, perceptions and practices in the French general population: The room for improvement. BMC Public Health. 2013;13: 576 10.1186/1471-2458-13-576 23764171 PMC3849746

[44] Roya News. Jordan free from corona cases as infected citizen left quarantine. In: Roya News [Internet]. 13 Mar 2020 [cited 30 Mar 2020]. https://en.royanews.tv/news/20214/Jordan-free-from-corona-cases-as-infected-citizen-left-quarantine

[45] Ababneh M, Jaber M, Rababa’h A, Ababneh F. Seasonal influenza vaccination among older adults in Jordan: prevalence, knowledge, and attitudes. Hum Vaccines Immunother. Taylor and Francis Inc.; 2020; 10.1080/21645515.2020.1718438 32045332 PMC7553699

[46] Lee BY. No, COVID-19 Coronavirus Was Not Bioengineered. Here’s The Research That Debunks That Idea. In: Forbes [Internet]. 17 Mar 2020 [cited 30 Mar 2020]. https://www.forbes.com/sites/brucelee/2020/03/17/covid-19-coronavirus-did-not-come-from-a-lab-study-shows-natural-origins/#f4a2cd73728c

[47] Science Daily. COVID-19 coronavirus epidemic has a natural origin—ScienceDaily. In: Science Daily [Internet]. 17 Mar 2020 [cited 30 Mar 2020]. https://www.sciencedaily.com/releases/2020/03/200317175442.htm

[48] Lenthang M. Coronavirus did NOT come from a lab: Experts debunk myths that China or USA bioengineered COVID-19. Mail Online. 23 Mar 2020. https://www.dailymail.co.uk/news/article-8143023/Experts-debunk-myths-China-USA-bioengineered-COVID-19.html. Accessed 30 Mar 2020.

[49] Boni MF, Lemey P, Jiang X, Lam TT-Y, Perry BW, Castoe TA, et al Evolutionary origins of the SARS-CoV-2 sarbecovirus lineage responsible for the COVID-19 pandemic. Nat Microbiol. Nature Publishing Group; 2020; 1–10. 10.1038/s41564-020-0771-4 32724171

[50] Zhong B-L, Luo W, Li H-M, Zhang Q-Q, Liu X-G, Li W-T, et al Knowledge, attitudes, and practices towards COVID-19 among Chinese residents during the rapid rise period of the COVID-19 outbreak: a quick online cross-sectional survey. Int J Biol Sci. Ivyspring International Publisher; 2020;16: 1745–1752. 10.7150/ijbs.45221 32226294 PMC7098034

[51] WHO. Coronavirus disease 2019 (COVID-19) Situation Report– 51 [Internet]. 11 Mar 2020.

[52] Albarrak AI, Mohammed R, Al Elayan A, Al Fawaz F, Al Masry M, Al Shammari M, et al Middle East Respiratory Syndrome (MERS): Comparing the knowledge, attitude and practices of different health care workers. J Infect Public Health. Elsevier Ltd; 2019; 10.1016/j.jiph.2019.06.029 31431424 PMC7102554

